# Adverse effects of being underweight on young female breast cancer patients with lymph node metastases

**DOI:** 10.7150/jca.38567

**Published:** 2020-02-03

**Authors:** Bo Chen, Jianguo Lai, Liping Guo, Danian Dai, Rong Chen, Guangnan Wei, Ning Liao

**Affiliations:** 1 Department of Breast Cancer, Cancer Center, Guangdong Provincial People's Hospital and Guangdong Academy of Medical Sciences, Guangzhou, Guangdong, China; 2 Department of Gynecology and Obstetrics, The Fifth Affiliated Hospital of Sun Yat-Sen University, Zhuhai, Guangdong, 519000, China; 3 Department of Gynecologic Oncology, State Key Laboratory of Oncology in South China, Collaborative Innovation Center for Cancer Medicine, Sun Yat-Sen University Cancer Center, Guangzhou, Guangdong, 510060, China; 4 Department of Breast Oncology, Sun Yat-Sen University Cancer Center, State Key Laboratory of Oncology in South China, Collaborative Innovation Center for Cancer Medicine, Guangzhou, China

**Keywords:** breast cancer, underweight, body mass index, prognosis

## Abstract

**Background**: This study aimed to examine the effect of underweight in breast cancer.

**Methods**: We performed a retrospective analysis of 3891 female patients diagnosed with primary breast cancer (I-IV stages). Body mass index (BMI) defined by World Health Organization criteria as follow: Underweight (UW; BMI<18.5 kg/m^2^), normal weight (NW; BMI =18.5-24.9 kg/m^2^) and overweight or obese (OW; BMI≥25 kg/m^2^). We performed to evaluate the association between low BMI and clinical outcome in different age (18-40 years and over 40 years) breast cancer.

**Results**: In our study, about 7% patients suffer from being underweight and 25% patients suffer from being overweight. Underweight is more prominent in young age group. Although no relationship was found between the recurrence rate and being underweight (HR 1.467(95 % CI 0.940-2.291), P=0.092 for disease-free survival), multivariate regression analysis confirmed that low BMI was an independent overall survival (OS) prognostic factor in young patients (HR 1.610(95 % CI 1.028-2.523), P=0.037 for OS). Further analysis showed the prognostic significance of underweight only seen in young patients with axillary lymph node metastasis or III-IV stage patients.

**Conclusions**: Our results demonstrate the prognostic importance of low BMI in young breast cancer patients (under 40 years old) with lymph node metastases. The role of low BMI in breast cancer might depend on patients' age and clinical stage.

## Introduction

Body mass index (BMI), as an internationally recognized indicator, provides a simple numeric measure of a person's thickness or thinness 1. It is allowing researchers to discuss weight problems more objectively. For a long time, people pay more attention to the relationship between obesity and disease. Many studies have confirmed that obesity is related to the occurrence and prognosis of breast cancer 2-4. The current mainstream view in the field of breast cancer research is that obesity will increase the incidence of breast cancer, and affect the survival of postmenopausal women with breast cancer 5, 6. Although weight control can reduce some of the health problems caused by obesity, few people understand the impact of underweight (BMI<18.5 kg/m^2^) on health. On the other hand, in today's society there is a trend that more and more women, especially young women, to pursue slim or even excessive weight loss for beauty. Meanwhile, underweight remains a severe public health problem especially in south Asian and central and east Africa 7. In 2014, the prevalence of underweight in women in South Asian countries was still 24.0% 8. Breast cancer is the highest incidence of malignant tumors all over the world amongst women 9, 10. It is meaningful to explore the relation of low body mass index to the prognosis of breast cancer. However, in contrast to the considerable body of work on obesity, the impact of being underweight on breast cancer has not been adequately addressed. Although it is not documented that being underweight increases the risk of breast cancer 11, Marret et al. provided data concerning the possibility that low body mass index may be related to local recurrence of breast cancer after conservative treatment 12. Meanwhile, several studies have reported that underweight populations should be considered as a risk factor for recurrence and overall mortality of breast cancer compared with those of normal weight 13-15. Although those previous evidences suggest that low body mass index may be related poor prognosis in breast cancer, it is worth reminding that it may not apply to all groups and subtypes. Therefore, there are still many questions about low body mass index in breast cancer worth further discussion. For instance, the proportion of low body mass index in different age groups and whether low body mass index affects breast cancer patients of different ages are still worth a step closer study.

In this study, our objective was to elucidate above issues to some extent. World Health Organization criteria were used to define BMI categories. We collected over ten years of breast cancer patient data from the largest cancer center in southern China. A total of 3891 patients with stage I-IV breast cancer diagnosed between 2001 and 2011 were included to explore the distribution of low BMI in different age groups and prognosis influences.

## Patients and methods

### Study population

Female patients who diagnosed with breast cancer and received treatment in Sun Yat-sen University Cancer Center between January 1, 2001 and December 31, 2011 were retrospectively studied. A total of 3891 patients were identified based on the following conditions: female patients diagnosed with invasive carcinoma with data on body mass index and treatment information. Patients with history of other cancers were excluded. Written informed consent was obtained from each patient upon admission to hospital. Patient's characteristics and clinical features were collected from the electronic medical record system. The pathologic TNM stage was determined according to the American Joint Committee on Cancer Classification, 7th edition (http://www.cancerstaging.org). Estrogen receptors (ER), progesterone receptors (PR) and human epidermal growth factor receptor-2 (HER-2) were evaluated by immunohistochemistry. ER positive or PR positive when 1% or more of the cells stained positive. HER-2 positive was defined with immunohistochemical scores of 3+. This study was conducted in accordance with Helsinki declaration and approved by the Institute Research Ethics Committee of Sun Yat-sen University Cancer Center.

### Follow-up and endpoints

All patients were subjected to a follow-up program that included outpatient visits or telephone interviews every 3 to 6 months. The last follow-up date to confirm the final conditions was 7 April 2017. Prognosis was evaluated based on disease-free survival (DFS) and overall survival (OS). Death resulting from causes other than breast cancer was treated as censored.

### Statistical analyses

Patients were divided into two different age groups: Young (18-40 years), Middle and Old (over 40 years). Underweight (UW; BMI<18.5 kg/m^2^), normal weight (NW; BMI =18.5-24.9 kg/m^2^) and overweight or obese (OW; BMI≥25 kg/m^2^) defined by World Health Organization criteria. All statistical analyses were performed using the Statistical Product and Service Solutions version 23.0 (SPSS, Chicago, IL). The Chi-Square test and Fisher's exact test were used to compare characteristics of each group. Log-rank test were used to analyze the differences between survival curves. Cox univariate model, the Hazard Ratio (HR) and the 95% confidence intervals (95% CI) were estimated for variables. Multivariate analysis was performed the variables that were found to be significant in univariate analysis by using stepwise regression (forward selection). Statistical significance was set at *P* < 0.05.

## Results

### Clinicopathologic features of BMI groups

**Figure 1:**
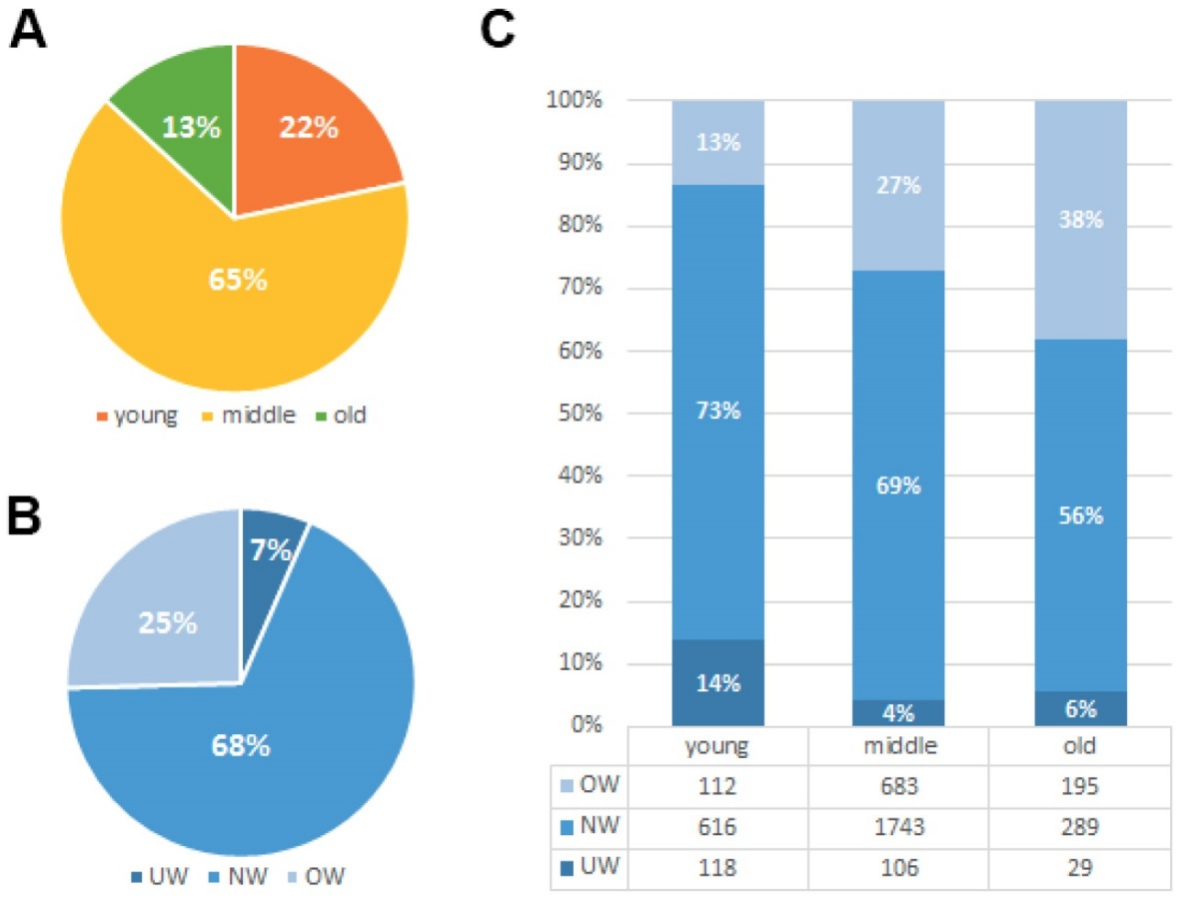
** Clinicopathologic features of BMI groups.** (A). Age-related rate of the 3891 enrolled breast cancer patients. (B). Constituent ratio of patients' BMI (Underweight (UW; BMI<18.5 kg/m^2^), normal weight (NW; BMI =18.5-24.9 kg/m^2^) and overweight or obese (OW; BMI≥25 kg/m^2^)). (C). Constituent ratio of patients' BMI in different age groups.

The median age for the 3891 enrolled patients was 48 (range: 18-92) years old. Their median follow-up time was 81 months (range, 1-197 months [censored]). Due to a long diagnosis time span and diverse TNM stages, patients received a great diversity of treatment modalities. Paclitexal-based combination chemotherapy, FEC (uorouracil/epirubicin/cyclophosphamide), CMF (cyclophosphamide/methotrexate/fluorouracil), EC (epirubicin/cyclophosphamide) and CAF/FAC (cyclophosphamide/doxorubicin/fluorouracil) were the main chemotherapy regimens 16. Of the 3891 patients included in this analysis, 616 (15.8%) patients have died and 624 (16.0%) patients have experienced a recurrence. As shown in Fig. 1A, the majority of patients in our study are less than 60 years' old (18-40years 22%, 40-60years 65% and over 60years 13%). Among those patients, about 7% patients suffer from underweight problem and 25% patients suffer from overweight problem when they underwent diagnosis of breast cancer (Fig. 1B). We found that weight problems are not the same in different age groups of patients. Underweight problems are mainly found in young patients, while overweight problems are getting worse with age (Fig. 1C). Meanwhile, body mass index classification showed a significant association with various prognostic factors such as age (*P*<0.001), menopause (*P*<0.001), tumor status (*P*<0.001), TNM staging (*P*=0.026) and histological grade (*P*=0.043) (Table 1). Furthermore, we investigated the relationship between BMI groups and clinicopathologic features in different ages groups (Table 2). We found that the results in the different age groups are different. In young age group, BMI classification showed a significant association with TNM staging (*P* = 0.018) and HER-2 status (*P* = 0.013). In middle and old age group, BMI classification showed a significant association with tumor status (*P* = 0.001).

### Recurrence and survival outcomes of the BMI groups

**Figure 2:**
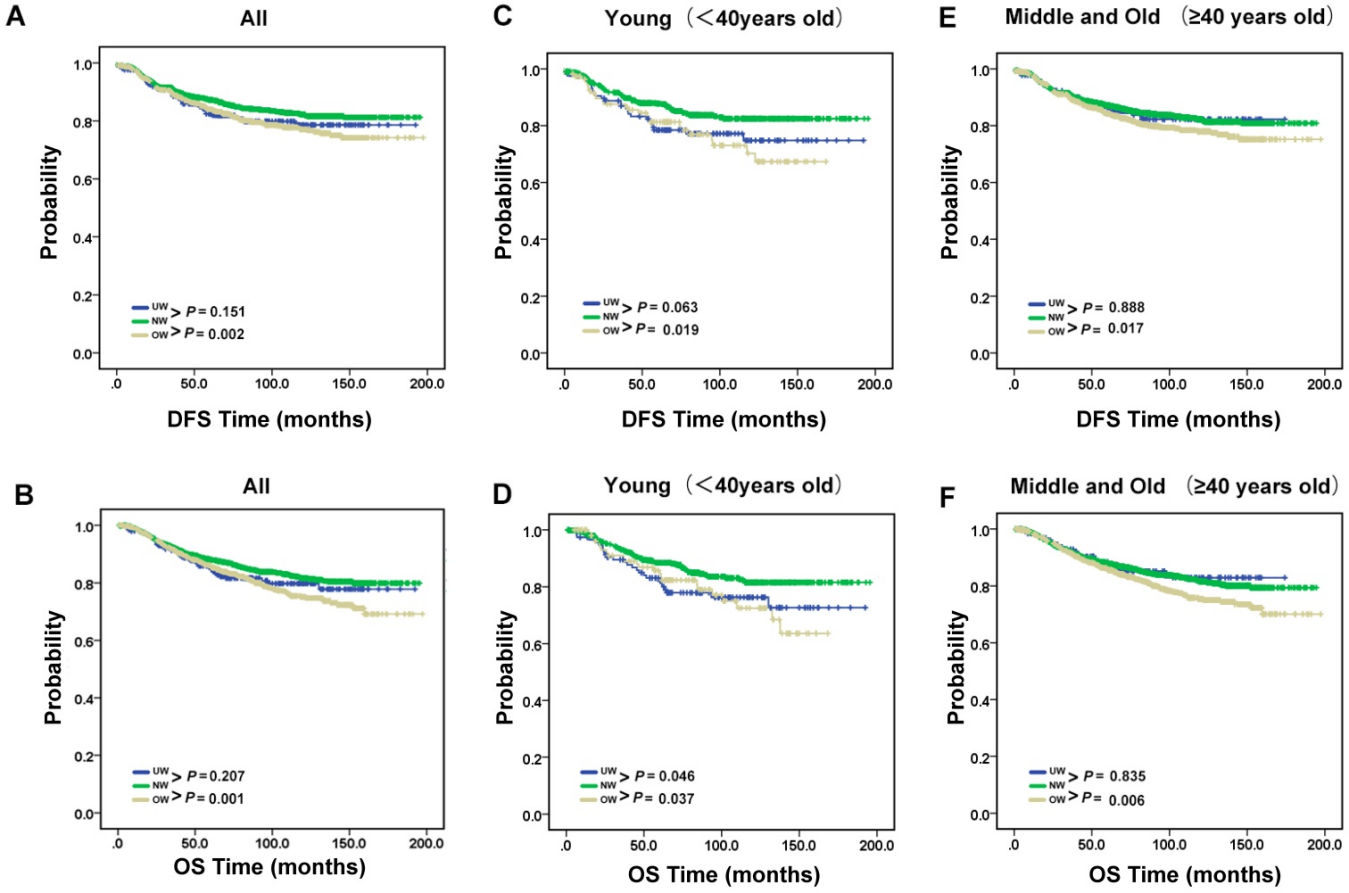
** Recurrence and survival outcomes of the BMI groups.** (A). DFS and (B). OS of the 3891 enrolled breast cancer patients. (C). DFS and (D). OS of young breast cancer patients. (E). DFS and (F). OS of middle and old breast cancer patients.

Next, we conducted a survival analysis to test how body mass index is related to clinical prognosis (Fig. 2A-B). The results showed that DFS and OS were significantly worse for overweight group patients compared with normal group patients (*P* = 0.002 for DFS; *P* = 0.001 for OS). However, compared with normal group patients, it is not obvious an interaction between underweight and DFS or OS (*P* = 0.151 for DFS; *P* = 0.207 for OS). Therefore, we used stratified analysis to explore the impact of underweight on breast cancer patients with different ages (Fig. 2C-F). The prognostic significance of OS of underweight were only seen in young age group patients (*P*=0.046). Meanwhile, the results of overweight group are also shown in the Fig. 2C-F.

## Discussion

Weight problems are rapidly becoming a vital health issue affecting many people in the world 17-19. Especially the consequences of the epidemic of overweightness or obesity have caused extensive concern amongst common people 20. Research shows that being overweight or obese is linked to a greater risk of some types of cancers including postmenopausal breast, colon, kidney, and esophageal cancers 6, 21, 22. On the other hand, being underweight is also a problem that can't be neglected 14. However, very few analyses of trends in being underweight 23, especially for cancer patients, and in severe and morbid obesity have been done. Meanwhile, many researchers included underweight patients in the normal reference group in previous studies 24, 25. Compared to overweightness or obesity, underweight does not arouse sufficient attention. In this study, we demonstrated in 3891 Chinese patients that being underweight (BMI<18.5 kg/m^2^) was associated with adverse outcomes in young patients (under 40 years old) with lymph node metastasis. We noticed that many previously studies excluded IV stage patients. Our study included all stages breast cancer patients which better reveals the real status. About 32% patients face weight problems. It followed a pattern with 25% of patients being either overweight or obese and 7% of patients categorized as being underweight. Meanwhile, we notice that the major weight problem will be different depending on diagnosis age. Underweight is prominent in young age group. While overweight is mainly in middle or old aged patients. This phenomenon might mirror recent trends in the Chinese population. It has been well documented that human race has a significant effect on the peak age of breast cancer 26. The peak age is between 60 and 70 years in Western countries, but is between 40 and 50 years in Asian countries 27. Therefore, we have more premenopausal and young patients. In a German study which involved more than 8,800 breast cancer patients show that only 1.5% (134/8,872) of patients were underweight, while about 50% of patients were overweight or obese^28^. Meanwhile, a New Zealand study showed that for 5,458 new breast cancers, BMI was normal (18.5-24.9kg/m^2^) for 32.7%, overweight (25-29.9kg/m^2^) 31.1%, obese (>30kg/m^2^) 34.9% and 1.3% underweight (<18.5kg/m^2^) 29. Thus, our results suggested that underweight problem might be more widespread in Asian breast cancer.

By investigating the relationship between clinicopathological parameters and BMI, we found that BMI was significantly associated with age. However, previous studies didn't discuss the relationship between clinicopathological parameters and BMI by age. The stratified analysis showed that the results in different ages were not entirely identical. Meanwhile, we did not find the relationship between BMI and distant metastasis, which is consistent with the previous study. A recent study screening 848 primary operable breast cancer patients demonstrated that normal weight (BMI = 18-24.9 kg/m^2^) patients were more likely to present with HER-2+ breast cancer and overweight (BMI = 25-29.9 kg/m^2^) and obese (BMI > 30 kg/m^2^) patients with TNBC 30. Nevertheless, we failed to found such an association between BMI and breast cancer subtype. The differences results might be due to race, BMI classification criteria, numbers of patients and inclusion criteria. We believe a correlation between HER-2+ and BMI needs more studies to confirm, while we found a statistics significance (P=0.013) in young patients. Because in our study, there were a lot of HER-2 (2+) patients who didn't receive FISH to determine the HER-2 statue. Besides, when we focused on underweight, we found the most underweight patients in I-II staging, suggesting that underweight wasn't limited in breast cancer end-stage.

A growing body of evidence exists linking BMI to prognostic outcome among female breast cancer patients 3, 31, 32. In this study, we observed this relationship linking was a little complex. Age, lymph node status and TNM staging could affect it. The results confirmed underweight prognosis value of OS in young age group. Low BMI in young female breast cancer patients with lymph node metastases predict adverse prognosis. For a long time doctors wanted to give patients the best treatment but ignored the nutrition support. So far, there was no large-scale survey data about cancer patient nutrition status. Based on our results, we believe that for underweight young breast cancer patients with lymph node metastasis, we should put more attention on nutrition support in their treatment. Although the biological mechanisms are not fully understood, nutrition support might largely improve the prognosis of this cohort of patients. There are several possible mechanisms may explain the worse prognosis of underweight patients. First, underweight patients have decreased physiologic reserve which may make them more vulnerable to adverse events 33. Second, underweight patients were less tolerant of cancer therapies and at higher risk of procedure-related complications. Finally, some BMI-associated risk factors and comorbidities may be more prevalent in underweight patients such as malnutrition, inflammatory and autoimmune disorders, and multi-organ dysfunction. Malnutrition due to being underweight may compromise immune function and surveillance. But, categories of underweight may include both active healthy patients with an inherited lean body type and undernourished patients. Therefore, the detailed remains to be further explored. There are several limitations including the retrospective nature of our study. First, we didn't have data on weight changes. Moreover, all of patients in our study are Chinese and we lacked information on BMI-associated risk factors and comorbidities which might have an influence on clinical outcomes. Finally, the number of underweight patients was relatively small, although our sample size was large.

## Conclusions

To our knowledge, this is the first Chinese large single-institution retrospective study to assess the association between prognostic outcome and low BMI among female breast cancer with stage I-IV. Our results indicate that underweight problem might be more widespread in Asian breast cancer and prominent in young age group. There is a clear prognostic value of OS of low BMI at the time of initial diagnoses among young female breast cancer patients with lymph node metastases. While no relationship was found between the recurrence rate and underweight. The role of low BMI in breast cancer might depend on patients' clinical characteristics such as lymph node status and advanced stage. Supplementary nutrition support might be improving advanced stage underweight young female breast cancer patients' outcome. Future research is needed to confirm whether underweight is unrelated to breast cancer subtypes.

## Figures and Tables

**Figure 3:**
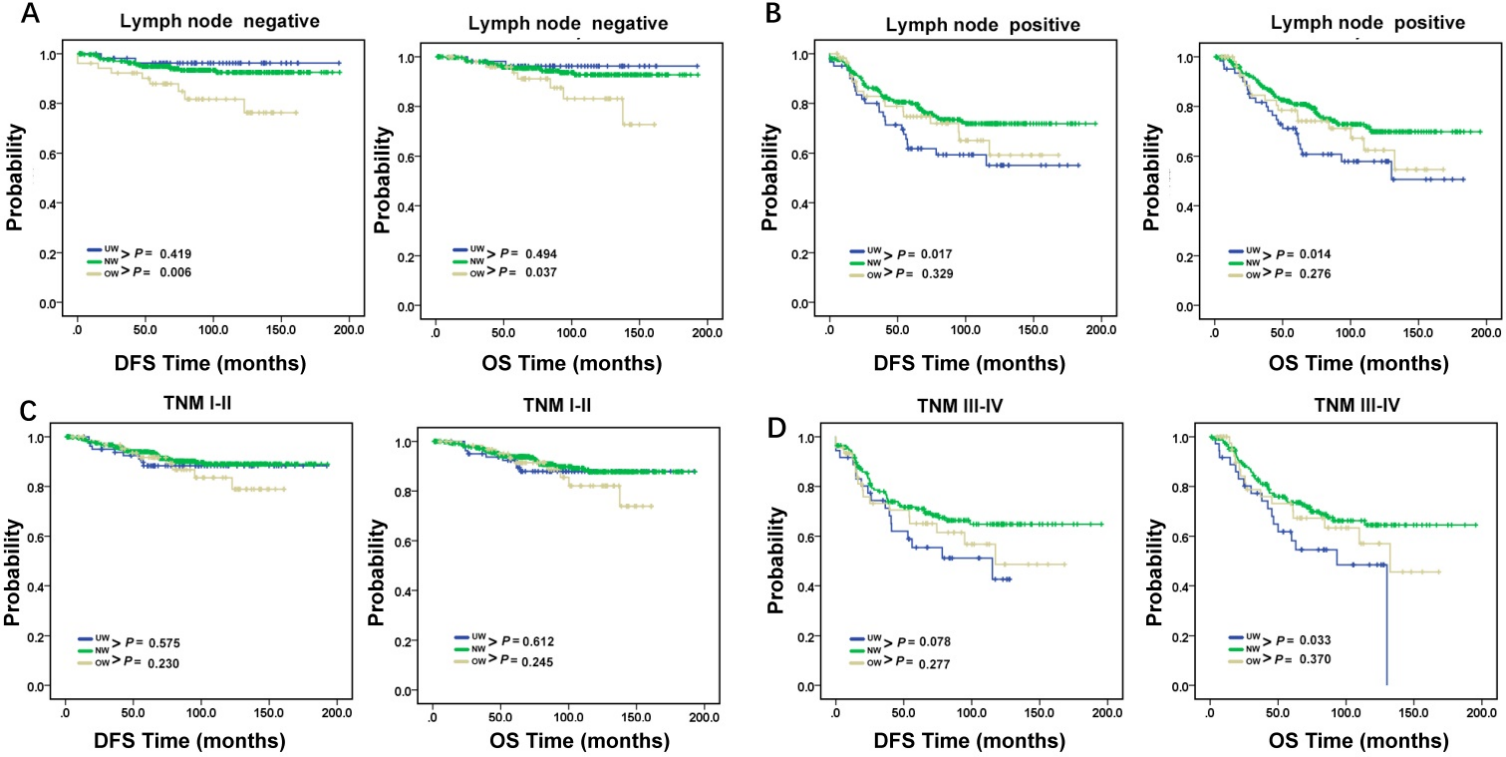
** DFS and OS in young breast cancer patients.** DFS and OS of young breast cancer patients with (A). lymph node negative and (B). lymph node positive. DFS and OS of young breast cancer patients with (C). TNM stage I-II and (D). TNM stage III-IV. *P* < 0.05, statistically significant.

**Table 1:** Correlation between BMI and clinicopathological variables of breast cancer patients.

Variables	Cases (n= 3891)	SYSUCC Data Set						P value
		UW		NW		OW		
		No.	%	No.	%	No.	%	
Age (years)								<0.001
<40	846	118	46.6%	616	23.3%	112	11.3%	
≥40	3045	135	53.4%	2032	76.7%	878	88.7%	
Menopause								<0.001
No	2380	189	74.7%	1727	65.2%	464	46.9%	
Yes	1511	64	25.3%	921	34.8%	526	53.1%	
Tumor status (T)								<0.001
T1	1117	78	30.8%	819	30.9%	220	22.2%	
T2	2134	134	53.0%	1419	53.6%	581	58.7%	
T3	369	20	7.9%	241	9.1%	108	10.9%	
T4	271	21	8.3%	169	6.4%	81	8.2%	
Axillary lymph node metastasis								0.364
No	1961	135	53.4%	1343	50.7%	483	48.8%	
Yes	1930	118	46.6%	1305	49.3%	507	51.2%	
Distance metastasis (M)								0.749
No	3756	244	96.4%	2560	96.7%	952	96.2%	
Yes	135	9	3.6%	88	3.3%	38	3.8%	
TNM Staging								0.026
I-II	2806	185	73.1%	1940	73.3%	681	68.8%	
III-IV	1085	68	26.9%	708	26.7%	309	31.2%	
Histological grade								0.043
G1	122	7	2.8%	91	3.4%	24	2.4%	
G2	1932	137	54.2%	1333	50.3%	462	46.7%	
G3	1837	109	43.1%	1224	46.2%	504	50.9%	
ER status								0.757
Negative	1549	105	41.5%	1045	39.5%	399	40.3%	
Positive	2264	143	56.5%	1554	58.7%	567	57.3%	
Unknown	78	5	2.0%	49	1.9%	24	2.4%	
PR status								0.630
Negative	1471	102	40.3%	987	37.3%	382	38.6%	
Positive	2348	146	57.7%	1616	61.0%	586	59.2%	
Unknown	72	5	2.0%	45	1.7%	22	2.2%	
HER-2 status								0.224
- or +	2516	154	60.9%	1720	65.0%	642	64.8%	
++	353	29	11.5%	248	9.4%	76	7.7%	
+++	766	50	19.8%	503	19.0%	213	21.5%	
Unknown	256	20	7.9%	177	6.7%	59	6.0%	
Subtype								0.397
Luminal A or B	2667	169	66.8%	1828	69.0%	670	67.7%	
HER-2	425	30	11.9%	273	10.3%	122	12.3%	
TNBC	718	48	19.0%	498	18.8%	172	17.4%	
Unknown	81	6	2.4%	49	1.9%	26	2.6%	

* P < 0.05, statistically significant.

**Table 2:** The relationship between BMI groups and clinicopathologic features in different ages groups.

Variables	Young						P value	Middle and Old						P value
	UW		NW		OW			UW		NW		OW		
	No.	%	No.	%	No.	%		No.	%	No.	%	No.	%	
Tumor status (T)							0.168							0.001
T1	41	34.7%	197	32.0%	24	21.4%		37	27.4%	622	30.6%	196	22.3%	
T2	57	48.3%	311	50.5%	61	54.5%		77	57.0%	1108	54.5%	520	59.2%	
T3	10	8.5%	70	11.4%	19	17.0%		10	7.4%	171	8.4%	89	10.1%	
T4	10	8.5%	38	6.2%	8	7.1%		11	8.1%	131	6.4%	73	8.3%	
Axillary lymph node metastasis							0.600							0.168
No	57	48.3%	315	51.1%	52	46.4%		78	57.8%	1028	50.6%	431	49.1%	
Yes	61	51.7%	301	48.9%	60	53.6%		57	42.2%	1004	49.4%	447	50.9%	
Distance metastasis (M)							0.343a							0.374a
No	111	94.1%	596	96.8%	108	96.4%		133	98.5%	1964	96.7%	844	96.1%	
Yes	7	5.9%	20	3.2%	4	3.6%		2	1.5%	68	3.3%	34	3.9%	
TNM Staging							0.018							0.092
I-II	82	69.5%	445	72.2%	66	58.9%		103	76.3%	1495	73.6%	615	70.0%	
III-IV	36	30.5%	171	27.8%	46	41.1%		32	23.7%	537	26.4%	263	30.0%	
Histological grade							0.378a							0.101
G1	2	1.7%	25	4.1%	2	1.8%		5	3.7%	66	3.2%	22	2.5%	
G2	62	52.5%	301	48.9%	49	43.8%		75	55.6%	1032	50.8%	413	47.0%	
G3	54	45.8%	290	47.1%	61	54.5%		55	40.7%	934	46.0%	443	50.5%	
ER status							0.275a							0.639
Negative	47	39.8%	242	39.3%	56	50.0%		58	43.0%	803	39.5%	343	39.1%	
Positive	69	58.5%	359	58.3%	53	47.3%		74	54.8%	1195	58.8%	514	58.5%	
Unknown	2	1.7%	15	2.4%	3	2.7%		3	2.2%	34	1.7%	21	2.4%	
PR status							0.953a							0.595
Negative	45	38.1%	218	35.4%	41	36.6%		57	42.2%	769	37.8%	341	38.8%	
Positive	71	60.2%	285	46.3%	68	60.7%		75	55.6%	1231	60.6%	518	59.0%	
Unknown	2	1.7%	13	2.1%	3	2.7%		3	2.2%	32	1.6%	19	2.2%	
HER-2 status							0.013							0.304
- or +	74	62.7%	413	67.0%	71	63.4%		80	59.3%	1307	64.3%	571	65.0%	
++	17	14.4%	52	8.4%	2	1.8%		29	21.5%	395	19.4%	185	21.1%	
+++	21	17.8%	108	17.5%	28	25.0%		12	8.9%	196	9.6%	74	8.4%	
Unknown	6	5.1%	43	7.0%	11	9.8%		14	10.4%	134	6.6%	48	5.5%	
Subtype							0.565							0.550
Luminal A or B	81	68.6%	419	68.0%	70	62.5%		17	12.6%	221	10.9%	106	12.1%	
HER-2	13	11.0%	52	8.4%	16	14.3%		88	65.2%	1409	69.3%	600	68.3%	
TNBC	22	18.6%	131	21.3%	23	20.5%		26	19.3%	367	18.1%	149	17.0%	
Unknown	2	1.7%	14	2.3%	3	2.7%		4	3.0%	35	1.7%	23	2.6%	

* P < 0.05, statistically significant. a. using Fisher's exact test

**Table 3:** Univariate and Multivariate COX regression analysis for Disease-free Survival and Overall Survival in in young age breast cancer patient group.

Variables	Disease-free Survival				Overall Survival			
	Univariate analysis		Multivariate analysis		Univariate analysis		Multivariate analysis	
	HR(95%CI)	p value	HR(95%CI)	p value	HR(95%CI)	p value	HR(95%CI)	p value
TNM Staging								
I	1 (Reference)		1 (Reference)		1 (Reference)		1 (Reference)	
II	1.837(0.950-3.552)	0.071	1.525(0.783-2.972)	0.215	2.057(1.038-4.075)	0.039	1.756(0.881-3.503)	0.110
III	5.631(2.965-10.695)	<0.001	3.999(2.071-7.721)	<0.001	6.263(3.211-12.216)	<0.001	4.241(2.137-8.418)	<0.001
IV	27.496(13.363-56.575)	<0.001	15.965(7.477-34.088)	<0.001	22.081(10.088-48.333)	<0.001	11.317(4.969-25.777)	<0.001
Histological grade								
G1	1 (Reference)		1 (Reference)		1 (Reference)		1 (Reference)	
G2	2.683 (0.368-19.558)	0.330	1.978 (0.268-14.582)	0.503	2.552(0.349-18.651)	0.356	1.853 (0.251-13.704)	0.546
G3	8.184 (1.142-58.657)	0.036	3.788 (0.518-27.694)	0.189	8.031(1.120-57.578)	0.038	3.955 (0.541-28.938)	0.176
ER status (Negative vs Positive)	0.622(0.441-0.876)	0.007	0.809 (0.566-1.155)	0.244	0.605(0.426-0.861)	0.005	0.784(0.484-1.156)	0.191
PR status (Negative vs Positive)	0.732(0.518-1.036)	0.078	-	-	0.697 (0.489-0.992)	0.045	1.063(0.688-1.642)	0.782
HER-2 status								
- or +	1 (Reference)		1 (Reference)		1 (Reference)		1 (Reference)	
++	1.018(0.543-1.909)	0.955	0.939 (0.492-1.791)	0.848	0.963 (0.499-1.860)	0.911	0.804 (0.410-1.575)	0.525
+++	1.304(1.034-2.276)	0.033	1.124 (0.749-1.686)	0.573	1.604 (1.077-2.388)	0.020	1.185 (0.785-1.788)	0.420
BMI								
Normal weight	1 (Reference)		1 (Reference)		1 (Reference)		1 (Reference)	
Underweight	1.501(0.970-2.324)	0.068	1.467(0.940-2.291)	0.092	1.557(1.003-2.416)	0.048	1.610(1.028-2.523)	0.037
Overweight	1.676(1.083-2.595)	0.021	1.457(0.931-2.281)	0.099	1.611 (1.024-2.535)	0.039	1.383(0.871-2.196)	0.169

*Statistically significant prognostic factor identified by Univariate/Multivariate analysis

## Abbreviations

BMI: body mass index
DFS: disease-free survival
ER: estrogen receptors
HER-2: human epidermal growth factor receptor-2
PR: progesterone receptors
NW: normal weight
OS: overall survival
OW: overweight
TNM: Tumor, Node and Metastasis
UW: underweight

## References

[1] Martin L, Birdsell L, Macdonald N, Reiman T, Clandinin MT, McCargar LJ (2013). Cancer cachexia in the age of obesity: skeletal muscle depletion is a powerful prognostic factor, independent of body mass index. Journal of clinical oncology: official journal of the American Society of Clinical Oncology.

[2] Jiralerspong S, Goodwin PJ (2016). Obesity and Breast Cancer Prognosis: Evidence, Challenges, and Opportunities. Journal of clinical oncology: official journal of the American Society of Clinical Oncology.

[3] Arnold M, Jiang L, Stefanick ML, Johnson KC, Lane DS, LeBlanc ES (2016). Duration of Adulthood Overweight, Obesity, and Cancer Risk in the Women's Health Initiative: A Longitudinal Study from the United States. PLoS medicine.

[4] Park J, Morley TS, Kim M, Clegg DJ, Scherer PE (2014). Obesity and cancer-mechanisms underlying tumour progression and recurrence. Nature reviews Endocrinology.

[5] Neuhouser ML, Aragaki AK, Prentice RL, Manson JE, Chlebowski R, Carty CL (2015). Overweight, Obesity, and Postmenopausal Invasive Breast Cancer Risk: A Secondary Analysis of the Women's Health Initiative Randomized Clinical Trials. JAMA oncology.

[6] Bhaskaran K, Douglas I, Forbes H, dos-Santos-Silva I, Leon DA, Smeeth L (2014). Body-mass index and risk of 22 specific cancers: a population-based cohort study of 5.24 million UK adults. Lancet.

[7] Collaboration NCDRF (2016). Trends in adult body-mass index in 200 countries from 1975 to 2014: a pooled analysis of 1698 population-based measurement studies with 19.2 million participants. Lancet.

[8] Kim JH, Yoon KH, Hur H, Park S, Kim JY, Park HS (2019). Prevalence of breast cancer-related risk factors in underweight premenopausal women: the Korea National Health and Nutrition Examination Survey IV-VI. Breast cancer research and treatment.

[9] Harbeck N, Gnant M (2017). Breast cancer. Lancet.

[10] Chen B, Tang H, Chen X, Zhang G, Wang Y, Xie X (2019). Transcriptomic analyses identify key differentially expressed genes and clinical outcomes between triple-negative and non-triple-negative breast cancer. Cancer management and research.

[11] Guo L, Li N, Wang G, Su K, Li F, Yang L (2014). [Body mass index and cancer incidence:a prospective cohort study in northern China]. Zhonghua liu xing bing xue za zhi = Zhonghua liuxingbingxue zazhi.

[12] Marret H, Perrotin F, Bougnoux P, Descamps P, Hubert B, Lefranc T (2001). Low body mass index is an independent predictive factor of local recurrence after conservative treatment for breast cancer. Breast cancer research and treatment.

[13] Moon HG, Han W, Noh DY (2009). Underweight and breast cancer recurrence and death: a report from the Korean Breast Cancer Society. Journal of clinical oncology: official journal of the American Society of Clinical Oncology.

[14] Flegal KM, Graubard BI, Williamson DF, Gail MH (2005). Excess deaths associated with underweight, overweight, and obesity. Jama.

[15] Kawai M, Tomotaki A, Miyata H, Iwamoto T, Niikura N, Anan K (2016). Body mass index and survival after diagnosis of invasive breast cancer: a study based on the Japanese National Clinical Database-Breast Cancer Registry. Cancer medicine.

[16] Chen B, Dai D, Tang H, Ai X, Chen X, Zhang X (2016). Pretreatment Hematocrit Is Superior to Hemoglobin as a Prognostic Factor for Triple Negative Breast Cancer. PloS one.

[17] Lavie CJ, De Schutter A, Milani RV (2015). Healthy obese versus unhealthy lean: the obesity paradox. Nature reviews Endocrinology.

[18] Berrington de Gonzalez A, Hartge P, Cerhan JR, Flint AJ, Hannan L, MacInnis RJ (2010). Body-mass index and mortality among 1.46 million white adults. The New England journal of medicine.

[19] Zheng W, McLerran DF, Rolland B, Zhang X, Inoue M, Matsuo K (2011). Association between body-mass index and risk of death in more than 1 million Asians. The New England journal of medicine.

[20] Moley KH, Colditz GA (2016). Effects of obesity on hormonally driven cancer in women. Science translational medicine.

[21] Lagergren J (2011). Influence of obesity on the risk of esophageal disorders. Nature reviews Gastroenterology & hepatology.

[22] Li R, Grimm SA, Chrysovergis K, Kosak J, Wang X, Du Y (2014). Obesity, rather than diet, drives epigenomic alterations in colonic epithelium resembling cancer progression. Cell metabolism.

[23] Mamun AA, Finlay JE (2015). Shifting of undernutrition to overnutrition and its determinants among women of reproductive ages in the 36 low to medium income countries. Obesity research & clinical practice.

[24] Ewertz M, Gray KP, Regan MM, Ejlertsen B, Price KN, Thurlimann B (2012). Obesity and risk of recurrence or death after adjuvant endocrine therapy with letrozole or tamoxifen in the breast international group 1-98 trial. Journal of clinical oncology: official journal of the American Society of Clinical Oncology.

[25] Park YM, White AJ, Nichols HB, O'Brien KM, Weinberg CR, Sandler DP (2017). The association between metabolic health, obesity phenotype and the risk of breast cancer. International journal of cancer.

[26] Demicheli R, Retsky MW, Hrushesky WJ, Baum M, Gukas ID, Jatoi I (2007). Racial disparities in breast cancer outcome: insights into host-tumor interactions. Cancer.

[27] Leong SP, Shen ZZ, Liu TJ, Agarwal G, Tajima T, Paik NS (2010). Is breast cancer the same disease in Asian and Western countries?. World journal of surgery.

[28] Fontanella C, Lederer B, Gade S, Vanoppen M, Blohmer JU, Costa SD (2015). Impact of body mass index on neoadjuvant treatment outcome: a pooled analysis of eight prospective neoadjuvant breast cancer trials. Breast cancer research and treatment.

[29] Robinson B, Currie M, Phillips E, Dachs G, Strother M, Morrin H (2017). Body mass index (BMI): association with clinicopathological factors and outcome of women with newly diagnosed breast cancer in New Zealand. The New Zealand medical journal.

[30] Gershuni V, Li YR, Williams AD, So A, Steel L, Carrigan E (2017). Breast cancer subtype distribution is different in normal weight, overweight, and obese women. Breast cancer research and treatment.

[31] Dawood S, Broglio K, Gonzalez-Angulo AM, Kau SW, Islam R, Hortobagyi GN (2008). Prognostic value of body mass index in locally advanced breast cancer. Clinical cancer research: an official journal of the American Association for Cancer Research.

[32] Munsell MF, Sprague BL, Berry DA, Chisholm G, Trentham-Dietz A (2014). Body mass index and breast cancer risk according to postmenopausal estrogen-progestin use and hormone receptor status. Epidemiologic reviews.

[33] Bucholz EM, Krumholz HA, Krumholz HM (2016). Underweight, Markers of Cachexia, and Mortality in Acute Myocardial Infarction: A Prospective Cohort Study of Elderly Medicare Beneficiaries. PLoS medicine.

